# Comparison of glucose-6 phosphate dehydrogenase status by fluorescent spot test and rapid diagnostic test in Lao PDR and Cambodia

**DOI:** 10.1186/s12936-018-2390-6

**Published:** 2018-06-22

**Authors:** Gisela Henriques, Koukeo Phommasone, Rupam Tripura, Thomas J. Peto, Shristi Raut, Coco Snethlage, Im Sambo, Nou Sanann, Chea Nguon, Bipin Adhikari, Tiengkham Pongvongsa, Mallika Imwong, Lorenz von Seidlein, Nicholas P. Day, Nicholas J. White, Arjen M. Dondorp, Paul Newton, Benedikt Ley, Mayfong Mayxay

**Affiliations:** 1 Mahidol Oxford Tropical Medicine Research Unit, Faculty of Tropical Medicine, Mahidol University, Bangkok, Thailand; 2 Department of Life Science, Imperial College London, London, UK; 3 Lao-Oxford-Mahosot Hospital-Wellcome Trust Research Unit, Vientiane, Lao PDR; 4 Centre for Tropical Medicine and Global Health, Nuffield Department of Clinical Medicine, University of Oxford, Oxford, UK; 5 School of Medicine, Amsterdam University, Amsterdam, The Netherlands; 6 National Centre for Parasitology, Entomology and Malaria Control, Phnom Penh, Cambodia; 7 Savannakhet Provincial Station of Malariology, Parasitology and Entomology, Savannakhet, Savannakhet Province Lao PDR; 8 Department of Molecular Tropical Medicine and Genetics, Faculty of Tropical Medicine, Mahidol University, Bangkok, 10400 Thailand; 9 Menzies School of Health Research, Darwin, Australia; 10 Faculty of Postgraduate Studies, University of Health Sciences, Vientiane, Lao PDR

**Keywords:** Malaria, Glucose-6-phosphate dehydrogenase, Rapid diagnostic test, Southeast Asia

## Abstract

**Background:**

Glucose-6-phosphate dehydrogenase (G6PD) deficiency is the most common enzymopathy worldwide. Primaquine is the only licensed drug that effectively removes *Plasmodium vivax* hypnozoites from the human host and prevents relapse. While well tolerated by most recipients, primaquine can cause haemolysis in G6PD deficient individuals and is, therefore, underused. Rapid diagnostic tests (RDTs) could permit ascertainment of G6PD status outside of laboratory settings and hence safe treatment in remote areas. The performance of the fluorescent spot test (Trinity, Ireland; FST) and a G6PD RDT (Carestart, USA) against spectrophotometry were assessed.

**Methods:**

Participants were enrolled during cross-sectional surveys in Laos and by purposive sampling in Cambodia. FST and RDT were performed during village surveys and 3 mL of venous blood was collected for subsequent G6PD measurement by spectrophotometry.

**Results:**

A total of 757 participants were enrolled in Laos and 505 in Cambodia. FST and RDT performed best at 30% cut-off activity and performed significantly better in Laos than in Cambodia. When defining intermediate results as G6PD deficient, the FST had a sensitivity of 100% (95%CI 90–100) and specificity of 90% (95%CI 87.7–92.2) in Laos and sensitivity of 98% (94.1–99.6) and specificity of 71% (95%CI 66–76) in Cambodia (p < 0.001). The RDT had sensitivity and specificity of 100% (95%CI 90–100) and 99% (95%CI 97–99) in Laos and sensitivity and specificity of 91% (86–96) and 93% (90–95) in Cambodia (p < 0.001). The RDT performed significantly better (all p < 0.05) than the FST when intermediate FST results were defined as G6PD deficient.

**Conclusion:**

The interpretation of RDT results requires some training but is a good alternative to the FST.

*Trial registration* clinicaltrials.gov; NCT01872702; 06/27/2013; https://clinicaltrials.gov/ct2/show/NCT01872702

## Background

The glucose-6-phosphate dehydrogenase (G6PD) enzyme is an essential element of the pentose phosphate pathway (PPP), the only pathway for human red blood cells (RBC) to maintain the cells redox power [1, 2]. The gene coding for the enzyme is on the X—chromosome (Xq28); to date more than 185 G6PD clinically relevant variants have been described that result in varying degrees of reduced G6PD activity, collectively called G6PD deficiency (G6PDd) [3–5]. G6PDd is the most common enzymopathy worldwide with more than 400 million individuals affected [6]. Hemizygous males and homozygous females harbour homogenous RBC populations with varying degrees of enzyme deficiency which depend on the genotype [7]. In contrast heterozygous females have a mix of G6PD deficient and G6PD normal erythrocytes. The ratio of the two populations is as the result of embryonic random X-chromosome inactivation (lyonisation) [8, 9]. In most heterozygous females, the proportion of normal and deficient erythrocytes is balanced but in some heterozygous females the majority of erythrocytes are deficient resulting in false-normal qualitative tests [9].

G6PDd reduces the half-life of affected erythrocytes by increasing the cells susceptibility to oxidizing agents [10]; in the presence of strong oxidants G6PDd erythrocytes will haemolyse. The resulting haemolytic anaemia can be triggered through ingestion of a range of oxidizing agents including 8-aminoquinolines (e.g. primaquine) [11, 12]. Primaquine is the only drug currently licensed that effectively kills *Plasmodium vivax* hypnozoites, thereby preventing relapses and sustained morbidity and mortality in the human host [13]. A daily dose of primaquine (0.25–0.5 mg/kg) for 14 days tends to result in the radical cure of vivax malaria but can trigger haemolysis in G6PD deficient individuals. In contrast a single low dose primaquine (0.25 mg/kg) is safe, i.e. does not trigger haemolysis in G6PD deficient individuals and kills *Plasmodium falciparum* gametocytes the sexual, infectious life stage of the parasite which are not affected by most other anti-malarials [14]. The single low dose primaquine regimen has however no effect on *P. vivax* hypnozoites.

*Plasmodium vivax* infection and G6PD-deficiency are common in Asia including in Laos and Cambodia [15–17]. Probably related to fears of drug-induced haemolysis among patients with G6PD-deficiency, radical cure for *P. vivax* using primaquine is not widely available, even though the drug is included in national treatment guidelines [18]. This divergence between policy and practice has been observed in most malaria endemic countries [19]. Several promising qualitative and quantitative point of care G6PD testing devices are in development and entering the market [20–23]. Primaquine regimens such as the 8 week regimen (0.75 mg/kg) or more experimental regimens which incremental increase primaquine could be an alternative to achieve radical cure of vivax malaria. These regimens capitalize on the finite number of senescent erythrocytes susceptible to haemolysis which are replaced by reticulocytes and early erythrocytes with a much lower liability for haemolysis [19]. Whether such regimens are safe and accepted remains to be seen. *Plasmodium vivax* patients frequently suffer relapse and associated health risks, and also contribute to ongoing transmission [24]. Southeast Asian countries have pledged to eliminate vivax malaria by 2030. To achieve this ambitious goal the reliable diagnosis of G6PD deficiency could be critical [25, 26].

The gold standard for the diagnosis of G6PDd is quantitative spectrophotometry, a costly assay that requires a good laboratory infrastructure and cannot provide results within a time frame suitable to guide treatment [3, 27]. The most widely used G6PD diagnostic in the field is the qualitative fluorescent spot test (FST), presumably due to its low price of approximately 0.1–3 USD/test [28, 29]. The test requires basic laboratory infrastructure, can be challenging to process and interpret under field conditions, and has a processing time of at least 1 hour, most of which is required to dry the spots [30, 31]. Several qualitative G6PD lateral flow assays (G6PD RDTs) have been introduced to the market over the last years that are suitable for diagnosis at the point of care and show better operational characteristics than the FST, but at higher prices ranging from 1.5 USD to 25 USD/test [29].

The aims of this study were to evaluate the performance of the FST as well as the lateral flow G6PD RDT (Accessbio/Carestart; 65 Clyde Rd. Suite A, Somerset, NJ 08873, USA) against the gold standard spectrophotometry.

## Methods

Blood samples were collected in four villages in Savannakhet Province, Lao PDR and five villages in Battambang province, western Cambodia. Apart from being resident within the study area at the time of enrolment and age above 4 years no further enrolment criteria applied in Laos. In Cambodia villagers had been screened for G6PD activity using the FST in 2015. All villagers found to be G6PD deficient in 2015 were invited to participate along with a randomly selected sex- and age-matched control from the same village in 2016. After informed consent was received, local healthcare providers collected 3 mL of blood by venepuncture. Blood samples were transported in ethylenediaminetetraacetic acid (EDTA) treated tubes (BD Vacutainer, USA) in cold boxes and stored at 4 °C for a maximum of 48 h until laboratory analysis of G6PD activity.

### Laboratory procedures

G6PD deficiency was assessed by the FST (Trinity Biotech Plc, IDA Business Park, Bray, Co Wicklow, Ireland), the CareStart G6PD rapid diagnostic test (G6PD RDT) screening kit (Access Bio. Inc., New Jersey, USA), and by quantitative spectrophotometry (Trinity Biotech, Ireland). Spectrophotometry results were adjusted for individual haemoglobin levels (Hb) as measured by HemoCue^®^ (HemoCue Hb301; HemoCue AB, Ängelholm, Sweden) in the laboratory. All G6PD assays were performed independently and blinded to G6PD status of the participant.

### Trinity Biotech fluorescent spot test (FST)

A total of 5 μL of whole blood was added to 100 μL G-6-PDH substrate solution. A first aliquot was immediately spotted onto filter paper and the remaining solution incubated at room temperature. A second and a third drop of blood-substrate mixture were blotted after 5 and 10 min of incubation. The filter papers were air dried for approximately 30 min and subsequently read under UV light. A sample with normal enzyme activity showed moderate to strong fluorescence after 5 min, and strong fluorescence after 10 min. A sample with intermediate G6PD activity showed no or weak fluorescence after 5 min and moderate fluorescence after 10 min, while a deficient sample had very little or no fluorescence after 10 min. The FST test was performed along with normal, intermediate and deficient controls (Catalogue numbers G6888, G5029, and G5888, respectively, Trinity Biotech, Ireland). All results were read by two independent observers; when the interpretation of the readers was discordant, a third reader, blinded to the previous results was called in.

### CareStart G6PD rapid diagnostic test (G6PD RDT)

The CareStart G6PD deficiency RDT screening Kit (Catalogue number RGPM-02572) was used according to the manufacturer’s instructions. Briefly, 2 µL of whole blood were pipetted from the EDTA tube and added to the sample well followed by the addition of two drops of the assay buffer. All results were read after 10 min. Tests showing a distinct purple colour were interpreted as G6PD normal, tests showing no colour change, or a very faint purple colour were classified as deficient. Tests with no blood migration or incomplete blood migration were repeated. Tests were classified as invalid after failing to obtain a valid result in at least three attempts.

### Trinity Biotech quantitative G6PD test (spectrophotometry reference assay)

G6PD activity was measured by spectrophotometry in duplicate using the Trinity Biotech Assay (Kit no. 345-B; Trinity Biotech, Bray, Ireland). Normal, intermediate and deficient G6PD activity controls (Catalogue numbers G6888, G5029, and G5888, respectively) were run in duplicate at the beginning of each assay day. Sample testing was done if all three control values fell within a predefined activity range provided by the manufacturer. Duplicates for which the measurement values differed by more than 10% were rejected for the analysis and retested. For each measurement 10 μL whole blood or control were added to 1 mL G6PD assay solution and incubated at room temperature for 5 min. Then, 2 mL of G6PD substrate were added to the solution and mixed by inversion. One millilitre of the mixture was aliquoted into ultraviolet (UV)-transparent disposable cuvettes (Eppendorf UVette cells, Germany). A spectrophotometer (Shimadzu UV 1800 series, Shimadzu, Kyoto, Japan) was used to measure the change in absorbance at 340 nm over 5 min. Applying a formula provided by the manufacturer (Trinity) G6PD activity was calculated as U/dL and normalized by haemoglobin measurements performed on the same sample at the same time by a digital haemoglobin meter (Hemocue 201, Hemocue, Ängelholm, Sweden).

### Statistical analysis

Analysis was done using STATA version 14.0. (StataCorp, USA) and Excel (Microsoft Corp, USA). Categorical data were compared using McNemar’s test for correlated proportions, the Chi squared test, or Fisher’s exact test as appropriate. Test performance was calculated using standard formula [32]. In Laos 100% G6PD activity was defined by calculating the adjusted male median (AMM) [28]. In Cambodia the study population was purposively selected and 100% G6PD activity was defined as 11.8 U/gHb based on previous studies [16, 21]. G6PD deficiency (G6PDd) was then defined as any result below 10, 30 or 70% of the AMM. The FST and G6PD RDT were evaluated considering spectrophotometry as the reference method. Areas under the receiver operating curve (ROC) were calculated for each assay applying 10, 30 and 70% cut-off activities and using the formula for binary tests [33], areas were subsequently compared for significant differences in size [34]. Analysis for the FST was done repeatedly, considering intermediate results (FSTdefint) either as G6PD deficient (FSTdef) or G6PD normal.

## Results

In Laos a total of 757 participants and in Cambodia 505 participants were enrolled during 2015–2016 and tested by all three diagnostic tests (FST, G6PD-RDT and spectrophotometry). Four G6PD-RDT results from Laos (0.5%) and seven from Cambodia (1.4%) were considered invalid and were not included in the analysis.

### Prevalence of G6PD deficiency

**Fig. 1:**
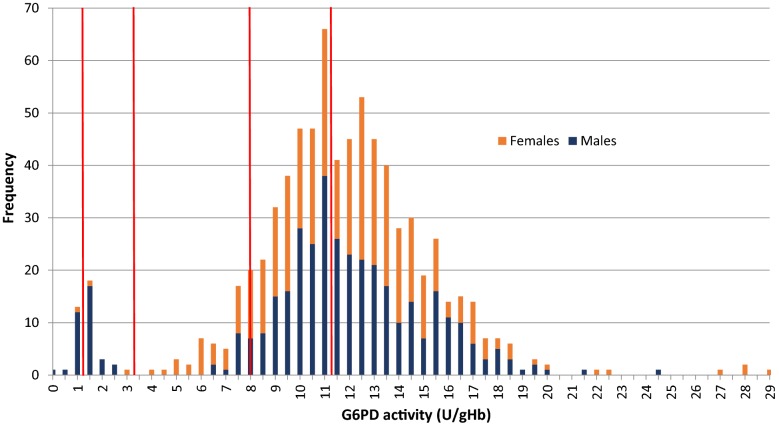
G6PD activity distribution by spectrophotometry in Laos. Red lines indicate 10, 30, 70 and 100% G6PD activity of the adjusted male median

The AMM by spectrophotometry (AMM) in Laos was 11.5 U/gHb, with 1.5% (n = 11) of all participants having G6PD activities below 10% of the AMM, 5.2% (n = 28) between 10 and below 30% and 12.2% (n = 53) having activities between 30 and below 70% of the AMM. A total of three female participants had G6PD activities below 30% of the AMM, compared to 36 male participants (p < 0.001), whereas significantly more females (n = 37) than males (n = 16) had intermediate G6PD activities between 30 and 70% of the AMM (Fig. 1). In the non-randomly selected Cambodian population (n = 505), 100% G6PD activity was defined as 11.8 U/gHb [16, 21], with 8.7% (n = 44) of all participants having G6PD activities of less than 10, 20.0% (n = 101) having G6PD activities between 10% and less than 30% G6PD activity and 28.3% (n = 143) having G6PD activities between 30% and less than 70% G6PD activity. Significantly less females than males had G6PD activities below 30% (n = 36, p < 0.001), whereas significantly more females had intermediate G6PD activities between 30% and 70% (n = 103) compared to males (p < 0.001).

### Test performance

Sensitivity and specificity of the FST and the G6PD RDT compared to spectrophotometry are presented in Table 1. Both FST and RDT performed significantly better in Laos compared to Cambodia, irrespective of definition or cut-off applied (Table 1).

**Table 1:** Test performance/cut-off activity/country

	Laos			Cambodia			
	Sensitivity (95%CI) (TP/TP+FN)	Specificity (95%CI) (TN/TN+FP)	ROC area (95%CI)	Sensitivity (95%CI) (TP/TP+FN)	Specificity (95%CI) (TN/TN+FP)	ROC area (95%CI)	p*
Cut-off activity = 10%							
FSTdefint	100.0% (71.5–100.0) (11/11)	86.73% (84.1–89.1) (647/746)	0.934 (0.921–0.946)	100.0% (92.0–100.0) (44/44)	56.2% (51.5–60.7) (259/461)	0.781 (0.758–0.804)	< 0.001
FSTdef	100.0% (71.5–100.0) (11/11)	96.25% (94.6–97.5) (718/746)	0.981 (0.974–0.988)	97.7% (88.0–99.9) (43/44)	80.5% (76.6–74.0) (371/461)	0.891 (0.862–0.920)	< 0.001
RDT	100.0% (71.5–100.0) (11/11)	94.88% (93.0–96.4) (704/742)	0.974 (0.966–0.982)	97.6% (87.1–99.9) (40/41)	75.3% (71.1–79.2) (344/457)	0.864 (0.833–0.895)	< 0.001
Cut-off activity = 30%							
FSTdefint	100.0% (90.1–100.0) (39/39)	90.1% (87.7–92.2) (647/718)	0.951 (0.940–0.961)	97.9% (94.1–99.6) (142/145)	71.1% (66.1–75.7) (256/360)	0.845 (0.819–0.871)	< 0.001
FSTdef	100.0% (90.1–100.0) (39/39)	100.0% (99.5–100.0) (718/718)	1.000 (1.000–1.000)	89.7% (83.5–94.1) (130/145)	99.2% (97.5–99.8) (357/360)	0.944 (0.920–0.969)	< 0.001
RDT	100.0% (90.1–100.0) (39/39)	98.6% (97.4–99.3) (704/714)	0.993 (0.989–0.997)	91.4% (85.5–95.5) (128/140)	93.0% (89.9–95.4) (333/358)	0.922 (0.895–0.949)	< 0.001
Cut-off activity = 70%							
FSTdefint	80.0% (67.7–89.2) (48/60)	91.1 (88.7–93.1) (635/697)	0.856 (0.803–0.908)	71.9% (66.3–77.0) (207/288)	82.0% (76.3–86.9) (178/217)	0.770 (0.733–0.806)	< 0.001
FSTdef	65.0% (51.6–76.8) (39/60)	100.0% (99.5–100.0) (697/697)	0.825 (0.764–0.886)	46.2% (40.3–52.1) (133/288)	100.0% (98.3–100.0) (217/217)	0.731 (0.702–0.760)	< 0.001
RDT	73.3% (60.3–83.9) (44/60)	99.3% (98.3–99.8) (688/693)	0.863 (0.807–0.920)	53.4% (47.3–59.3) (151/283)	99.1% (96.7–99.9) (213/215)	0.762 (0.732–0.792)	< 0.001

*FSTdefint* intermediate G6PD deficiency, *FSTdef*, G6PD deficiency, *RDT* rapid diagnostic test

*Derived from number of true and false results

#### Laos

**Fig. 2:**
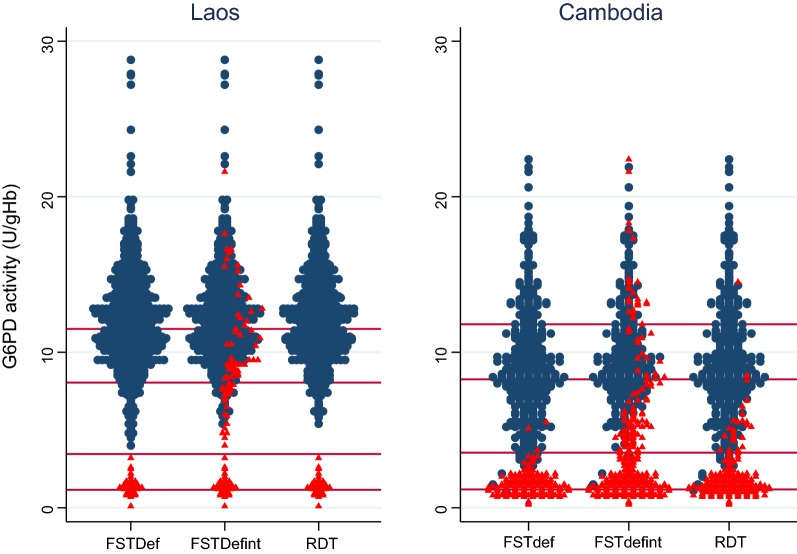
Distribution of G6PD normal and G6PD deficient test result by quantitative G6PD activity per assay and stratified by country. Red triangles = G6PD deficient test result, blue circles = G6PD normal test result. Red horizontal lines correspond to 10, 30, 70 and 100% G6PD activity of the AMM (from bottom to top), invalid RDT results are excluded

When intermediate results were considered as G6PD normal results and an AMM of 30% was considered as cut-off activity the FST showed a perfect match with spectrophotometry and performed significantly better (p < 0.001) than intermediate results defined as G6PD deficient (Table 1, Fig. 2). The G6PD RDT performed best at the same cut-off activity as the FST, while performance was lower than FSTdef (p = 0.002), the G6PD RDT performed significantly better than FSTdefint (p < 0.001) (Table 1).

#### Cambodia

Test performance of FSTdefint, FSTdef and the G6PD RDT was best at a cut-off activity of 30%. The FST performed significantly better when defining intermediate results as G6PD normal (p < 0.001) and FSTdef performed better than the G6PD RDT at a 30% cut-off (p < 0.001), while G6PD RDT performed significantly better than FSTdefint (p < 0.001) (Table 1). The FSTdef identified 15 (2.9%) and 1 (0.2%) samples and the G6PD RDT 12 (2.4%) and 1 (0.2%) samples as G6PD normal that had less than 30 and 10% of the AMM, respectively (Fig. 2). The false positives were not the same in the different test methods.

## Discussion

Despite suboptimal operational characteristics, the FST remains the most widely used G6PD diagnostic in Asia and possibly worldwide [28, 35]. Any test to replace the FST will need to perform at least comparable and ideally show better operational characteristics. While the FST showed a perfect match in Laos if a 30% cut-off activity was applied and intermediate results were categorized as G6PD normal. Applying a clinically appropriate approach and considering intermediate results G6PD deficient, the FST showed a sensitivity of above 95% in both countries, comparable to earlier reports from Asia [21, 27, 31, 36, 37]. The processing and interpretation of FSTs can be challenging under field conditions and this may be reflected in the lower performance of the test in Cambodia, where the laboratory technicians were less experienced than in Laos.

The G6PD RDT performed well in Laos at 30% cut-off activity and in both countries significantly better than FSTdefint. The results of the current study confirm earlier evaluation studies on the same version of the test [21, 31, 36, 37], suggesting good production standards. Considering test performance, its favourable operational characteristics and the tests current price of 1.5 USD/test (Carestart Accessbio, personal communication), the G6PD RDT is a good alternative to FST.

One of the key areas of deployment for qualitative G6PD diagnostics is to guide primaquine treatment, using a cut-off of 30% G6PD activity [3]. Tafenoquine, another 8-aminoquinoline is reaching registration and may be used in a single dose regimen for the radical cure of vivax malaria [38]. Tafenoquine will probably only be made available to patients with more than 70% G6PD activity and diagnostics with corresponding cut-off activities are needed. An accurate, affordable biosensor which can provide quantitative G6PD activity read-outs would be a major break-through in the management of *P. vivax* malaria as it would allow to customise 8-aminoquinoline therapy according to individual enzyme activity.

A major limitation of this study were the different selection criteria in Laos and Cambodia. While in Laos all eligible participants were tested by the diagnostic tests (FST, RDT, and spectrophotometry) in Cambodia only participants with a positive FST, along with village- sex-matched controls, were evaluated by RDT and spectrophotometry. Since the study population in Cambodia was not selected randomly, it was not possible to estimate the distribution of G6PD activity in the study population in Cambodia and define the prevalence of deficiency.

In conclusion, the G6PD RDT provides a good alternative to the widely used FST and both tests identified more than 90% of all G6PD deficient individuals not eligible for primaquine based radical cure. The RDT was sometimes difficult to interpret and needed to be repeated, therefore, careful training of health workers will be needed alongside deployment of these tests.

## Abbreviations

EDTA: ethylenediaminetetraacetic acid
FST: fluorescent spot test
FSTdef: G6PD deficient by FST
FSTdefint: intermediate G6PD deficiency by FST
G6PD: glucose 6 phosphate dehydrogenase
G6PDd: G6PD deficiency
Lao PDR: Lao Peoples Democratic Republic
NECHR: Cambodian National Ethics Committee for Health Research
OXTREC: Oxford Tropical Research Ethics Committee
RBC: red blood cell, erythrocyte
RDT: rapid diagnostic test
USA: United States of America
USD: United States Dollar
